# Statin regulated ERK5 stimulates tight junction formation and reduces permeability in human cardiac endothelial cells

**DOI:** 10.1002/jcp.26064

**Published:** 2017-08-03

**Authors:** Emma L. Wilkinson, James E. Sidaway, Michael J. Cross

**Affiliations:** ^1^ Department of Molecular and Clinical Pharmacology, MRC Centre for Drug Safety Science University of Liverpool Liverpool UK

**Keywords:** cardiotoxicity, ERK5, permeability, statin, ZO‐1

## Abstract

The MEKK3/MEK5/ERK5 signaling axis is required for cardiovascular development in vivo. We analyzed the physiological role of ERK5 in cardiac endothelial cells and the consequence of activation of this kinase by the statin class of HMG Co‐A reductase inhibitor drugs. We utilized human cardiac microvascular endothelial cells (HCMECs) and altered ERK5 expression using siRNA mediated gene silencing or overexpression of constitutively active MEK5 and ERK5 to reveal a role for ERK5 in regulating endothelial tight junction formation and cell permeability. Statin treatment of HCMECs stimulated activation of ERK5 and translocation to the plasma membrane resulting in co‐localization with the tight junction protein ZO‐1 and a concomitant reduction in endothelial cell permeability. Statin mediated activation of ERK5 was a consequence of reduced isoprenoid synthesis following HMG Co‐A reductase inhibition. Statin pretreatment could overcome the effect of doxorubicin in reducing endothelial tight junction formation and prevent increased permeability. Our data provide the first evidence for the role of ERK5 in regulating endothelial tight junction formation and endothelial cell permeability. Statin mediated ERK5 activation and the resulting decrease in cardiac endothelial cell permeability may contribute to the cardioprotective effects of statins in reducing doxorubicin‐induced cardiotoxicity.

## 1 INTRODUCTION

Endothelial cells form a monolayer on the luminal surface of the vasculature providing an interface that regulates the transcellular and paracellular passage of solutes and xenobiotics between circulation and tissues. Endothelial cell survival and proliferation is regulated by mitogen activated protein kinases (MAPKs) (Nithianandarajah‐Jones, Wilm, Goldring, Muller, & Cross, 2012, 2014). There are four major MAPKs: ERK1/2, JNK, P38, and ERK5. ERK5 is the least studied of the four major MAPKs, sharing 66% sequence homology with ERK2. However, ERK5 differs from other MAPKs with its extended C terminal tail containing a number of phosphorylation sites and transcriptional activation domain (Buschbeck & Ullrich, 2005). The ERK5 signaling axis has been demonstrated to be essential for cardiac development; knockout of *MEKK3/MEK5/ERK5* in mice is embryonically lethal at E9.5‐10.5 (Hayashi et al., 2004; Wang et al., 2005; Yang et al., 2000). Importantly, knockout of *ERK5* in adult mice leads to increased vascular leakage ultimately leading to death within 2–4 weeks (Hayashi et al., 2004). These data show that ERK5 activity is required in adults to preserve vascular integrity.

Endothelial junctions regulate the paracellular passage of ions and xenobiotics from the circulation to underlying tissues (Gonzalez‐Mariscal, Nava, & Hernandez, 2005). Endothelial cells express tight, adherens, and gap junctions (Dejana, Corada, & Lampugnani, 1995). These have been demonstrated to vary in isoform expression depending on their anatomical location. Tight junctions located within the brain microvasculature contain significantly higher levels of claudin 5 than in the glomerular vasculature, reflecting the difference in permeability between the vascular beds (Gunzel & Yu, 2013). Zonula occludens‐1 (ZO‐1) is a tight junction protein, which has recently been shown to regulate adherens junctions, angiogenesis, and endothelial barrier formation (Tornavaca et al., 2015). The endothelial barrier is altered in several diseases including peripheral edema (Dong, Li, Li, Shetty, & Fu, 2016), stroke, multiple sclerosis, and neurodegenerative diseases (Shi et al., 2016). In these conditions, changes in endothelial permeability allow water and solutes to permeate to underlying tissues, which leads to infiltration of immune cells (Shi et al., 2016). It is becoming apparent that anti‐cancer drugs are able to induce changes in endothelial permeability, which could be an initiating event in drug‐induced cardiotoxicity (Cross et al., 2015; Dong et al., 2016; Wolf & Baynes, 2006). We have recently shown that the anti‐cancer drugs doxorubicin and trastuzumab (Herceptin) can adversely affect cardiac microvascular endothelial tight junction formation leading to increased drug permeability (Wilkinson, Sidaway, & Cross, 2016).

ERK5 has been shown to be activated by a number of growth factors in a range of cell types (Nithianandarajah‐Jones et al., 2012). We have previously shown that in endothelial cells, VEGF stimulates ERK5 activity leading to increased cell survival (Roberts, Holmes, Muller, Cross, & Cross, 2010). Recent data have shown that vasoprotective drugs such as statins, which inhibit 3‐hydroxy‐3‐methyl‐glutaryl‐coenzyme A reductase (HMG‐CoA reductase) can also activate ERK5 in endothelial cells (Ohnesorge et al., 2010). In addition to their ability to lower LDL‐cholesterol, statins have several pleiotropic effects such as cardioprotection, however, the mechanism behind this is not yet fully understood (Chen et al., 2013; Henninger et al., 2012; Wang, Liu, & Liao, 2008). Statins competitively inhibit HMG‐CoA reductase (Stancu & Sima, 2001) preventing the production of cholesterol as well as isoprenoid intermediates such as farnesyl pyrophosphate (FPP) and geranylgeranyl pyrophosphates (GGPP) (Sen‐Banerjee et al., 2005). The ability of statins to ameliorate drug‐induced cardiovascular toxicity has recently been demonstrated in animal models, with cardioprotective effects attributed to effects on cardiomyocytes (Henninger et al., 2015; Yoshida, Shiojima, Ikeda, & Komuro, 2009).

Here, we report that statins activated ERK5 in human cardiac microvascular endothelial cells and that this activation results in an increase in tight junction formation and decreased permeability; statins can ultimately protect against doxorubicin injury of endothelial cells, which may contribute to the cardioprotective effects of these drugs.

## 2 MATERIALS AND METHODS

### 2.1 Cell culture

Human cardiac microvascular endothelial cells (HCMECs, C12285), human dermal microvascular endothelial cells (HDMECs, C‐12212), human coronary artery endothelial cells (HCAECs, C‐12221), human brain microvascular endothelial cells (HBMECs, C‐12287), human umbilical vein endothelial cells (HUVECs, C‐12205) were purchased from PromoCell (Heidelberg, Germany) and cultured as described previously (Wilkinson et al., 2016). A2780 cells were routinely cultured in RPMI 1640 medium (61870044, Gibco) supplemented with 10% (v/v) FCS. BT474 cells were routinely cultured in DMEM medium (D6429, Sigma, Poole, UK) supplemented with 10% (v/v) FCS. Cells were incubated at 37°C in humidified air containing 5% (v/v) CO_2_.

### 2.2 Cell stimulation and preparation of cell lysates

HCMECs were grown to confluence over 6 days in FGM with changes every 2–3 days, before addition of drugs. Simvastatin (S6196 purchased from Selleck Chemicals) was diluted to 0.3 μM in EBM supplemented with 1% (v/v) FCS and then added to cells for 6 hr either alone or as a pre‐incubation before addition of doxorubicin (S1208, Selleck Chemicals) diluted to 0.1 μM for a further 6 hr. BIX02189 (S1531, Selleck Chemicals) diluted to 1 μM was added 30 min prior to simvastatin treatment.

Cells were washed in ice‐cold DPBS on ice followed by addition of RIPA (20 mM Tris pH 7.5, 150 mM NaCl, 2.5 mM EDTA, 10% [w/v] glycerol, 1% [v/v] Triton X‐100; 1 mM Na_3_VO_4_; 10 μg/ml Aprotinin; 10 μg/ml Leupeptin; 10 μg/ml Pepstatin A; 1 mM PMSF; 0.5% [v/v] SDS; and 0.5% [v/v] sodium deoxycholate) lysis buffer. Lysates were centrifuged (17,000*g* for 20 min at 4°C) before diluting in LDS sample buffer (Invitrogen, Paisley, UK) containing 2‐mercaptoethanol (Sigma, Poole, UK) and denaturing at 90°C for 5 min.

### 2.3 Cell fractionation

Cellular fraction was performed using a Thermo Scientifc subcellular protein fractionation kit (78840). In brief, cells were harvested with trypsin‐EDTA and centrifuged [500*g* for 5 min]. All buffers contain the protease inhibitor cocktail provided with the kit. The cell pellet was washed in ice cold PBS and centrifuged [500*g* for 5 min]. The cell pellet was resuspended in cytoplasmic extraction buffer (CEB) and incubated on ice for 10 min. Lysates were centrifuged [500*g* for 5 min] and the supernatant transferred to a new tube for the cytoplasmic fraction. The pellet was resuspended in membrane extraction buffer (MEB) and incubated on ice for 10 min. Lysates were centrifuged [3,000*g* for 5 min] and the supernatant transferred to a new tube for the membrane fraction. The pellet was resuspended in nuclear extraction buffer (NEB) and incubated on ice for 30 min. Lysates for centrifuged [5,000*g* for 5 min] and the supernatant transferred to a new tube as the nuclear fraction. All fractions were diluted with LDS sample buffer containing 2‐mercaptoethanol and denatured at 90°C for 5 min.

### 2.4 Western blot and immunoprecipitation

For immunoprecipitation, lysates were incubated with ERK5 (2 μg/ml, #AF2848, R&D systems) or control IgG (2 μg/ml, goat) and protein G agarose. Lysates were separated by SDS–PAGE on 8% Tris‐glycine gels (8% [w/v] acrylamide, 0.4 M Tris–HCl pH 8.8 0.08% [w/v] SDS, 6.2% [v/v] glycerol 0.05% [v/v] temed, and 0.02% [w/v] APS). Gels were resolved for 90 min at 35 mA and transferred to 0.2 μm nitrocellulose membrane (Hybind‐C, GE Healthcare, Amersham, UK) for 2 hr at 125 mA. Membranes were blocked in 5% (w/v) bovine serum albumin (BSA) diluted in Tris‐buffered saline (TBS) with 0.1% (v/v) Tween‐20 (TBST) and probed with primary antibodies, directed against: ERK5 (#3372), phospho ERK1/2 (#4370), GAPDH (#5174), phospho ERK5 (T218/Y220) (#3371), ERK1/2 (#4695) were purchased from New England Biolabs (Hitchin, UK). Antibodies to RAP1 (SC‐65) and unprenylated RAP1A (SC‐1482) were purchased from Santa Cruz Biotechnology. Antibodies to MEKK2 (ab33918), MEKK3 (ab40750), and MEK5 (ab45146) were purchased from Abcam (Cambridge, UK). Antibody to ZO‐1 (40‐2200) was purchased from Invitrogen (Thermo Fisher Scientific). All antibodies were diluted in 1% (w/v) BSA and incubated overnight at 4°C. Proteins were detected using rabbit‐ or goat‐specific HRP secondary antibodies (Jackson Immunoresearch Labs) and enhanced‐chemiluminescence (ECL) Western blotting detection reagent (Pierce).

### 2.5 Immunofluorescence

HCMECs were fixed in 2% (w/v) PFA, permeabilized in 0.25% (v/v) triton X‐100 (for ZO‐1 and Alexa Fluor® Phalloidin 568 staining). For ERK5 staining cells were fixed in ice‐cold methanol at −20°C for 10 min. Cells were blocked in 1% (w/v) BSA diluted in TBST for 1 hr. Primary antibody and Alexa Fluor® secondary antibodies (Invitrogen, Thermo Fisher Scientific) were diluted in 1% (w/v) BSA diluted in TBST. Images were taken on a Zeiss AxioObserver Z1 inverted fluorescence microscope with Apotome2 optical sectioning device using an X63/1.25 oil immersion objective.

### 2.6 Trans‐endothelial permeability assay

HCMECs were plated onto ThinCerts™ 0.4 µm translucent (12‐well; Greiner, UK) and grown to confluence over 6 days. HCMECs were washed in DPBS before addition of 2 mg/ml FITC‐dextran 4 kDa (Sigma, Poole, UK) diluted in phenol red free EBM supplemented with 1% (v/v) FCS for 25 min at 37°C in a humidified 5% (v/v) CO_2_ atmosphere, after which the fluorescence level in the flow through was measured on a Varioskan plate reader (Ex 490 nm and Em 525 nm).

### 2.7 Trans‐endothelial electrical resistance (TEER) assay

HCMECs were plated onto ThinCerts™ 0.4 µm translucent (12‐well; Greiner, UK) and grown to confluence over 6 days. Resistance was measured using a Millicell ERS‐2 volt‐ohm meter (Millipore) and multiplied by the surface area of the insert to determine the TEER.

### 2.8 siRNA transfection

SiRNA duplexes were prepared as per the manufacturer's instructions (Non silencing D‐001810‐10‐05, MEKK2 L‐003582‐02‐0005, MEKK3 L‐003301‐00‐0005, MEK5 L‐003966‐00‐0005, ERK5 L‐003513‐00‐0005 smart pools, or ERK5 J‐003513‐08, J‐003513‐10 individual duplexes, Dharmacon, GE Healthcare, UK). HCMECs were transfected with 10 nM siRNA diluted in Opti‐MEM (Invitrogen, Thermo Fisher Scientific) with addition of 0.2% (v/v) Lipofectamine RNAiMAX for 6 hr. HCMEC were washed in DPBS with calcium and magnesium before addition of fresh FGM. Cells were allowed to grow to confluence for a further 6 days, with additional media changes every 2 days.

### 2.9 Adenoviral mediated gene expression

Constitutively active MEK5 cDNA (MEK5(D) HA‐Tag) and ERK5 wild‐type cDNA (ERK5 Flag‐Tag) (Roberts et al., 2010) were cloned into the adenoviral vector pAd‐DEST (Invitrogen). Recombinant adenovirus was generated by transient transfection of HEK293A cells and adenovirus purified using the Vivapure column (Sartorius, UK). HCMECs were infected with Ad‐CA‐MEK5 and Ad‐ERK5 at a MOI of 10 for 24 hr. Following transfection, cells were grown in endothelial FGM until confluent.

### 2.10 Viability assay

HCMECs, A2780s, and BT474s were plated on 96‐well gelatin‐coated plates and maintained at sub confluence for 24 hr. Cells were pre‐incubated with 0.3 μM simvastatin before addition of doxorubicin (30–0.001 μM) for 72 hr. Cells were lysed in Cell Titer Glo® (Promega, UK) as described previously (Wilkinson et al., 2016).

### 2.11 Biochemical addback procedure

HCMECs were cultured as described previously before addition of Squalene (#S3626), cholesterol (#C4951), mevalonolactone (#M4667), geranylgeranyl pyrophosphate (GGPP, #G6025), and farnesyl pyrophosphate ammonium salt (FPP, #F6892) (purchased from Sigma, Poole, UK), for 24 hr.

### 2.12 Quantitative real‐time PCR (qRT‐PCR) analysis

Total RNA was extracted from a range of endothelial cells and liver tissue using the RNAeasy kit following the manufacturer's instructions (Qiagen, Crawley, UK). cDNA synthesis was performed by reverse transcription of 1 μg total RNA as previously described (Wilkinson et al., 2016). Primers: *SLCO1B1* forward 5′ TTGGAGCTTGGTGGCTTAAT 3′; *SLCO1B1* reverse 5′ CCAGCACATGCAAAGACAGT 3′. qRT‐PCR was performed as described previously (Wilkinson et al., 2016). Cycle threshold (*C*_T_) values were determined for each mRNA sample and compared to the GAPDH control to determine gene expression changes using the comparative *C*_T_ (2^−ΔΔCT^) method (Livak & Schmittgen, 2001).

### 2.13 Statistical significance

This was performed using a one‐way analysis of variance (ANOVA) followed by Dunnett's post hoc test using SPSS software. Significant differences between control and test groups were evaluated with *p values ≤0.05 and **p values ≤0.01, indicated on the graphs. Error bars in graphs and histograms denote ± s.d. (standard deviation).

## 3 RESULTS

### 3.1 Endothelial tight junction formation is dependent on ERK5

**Figure 1:**
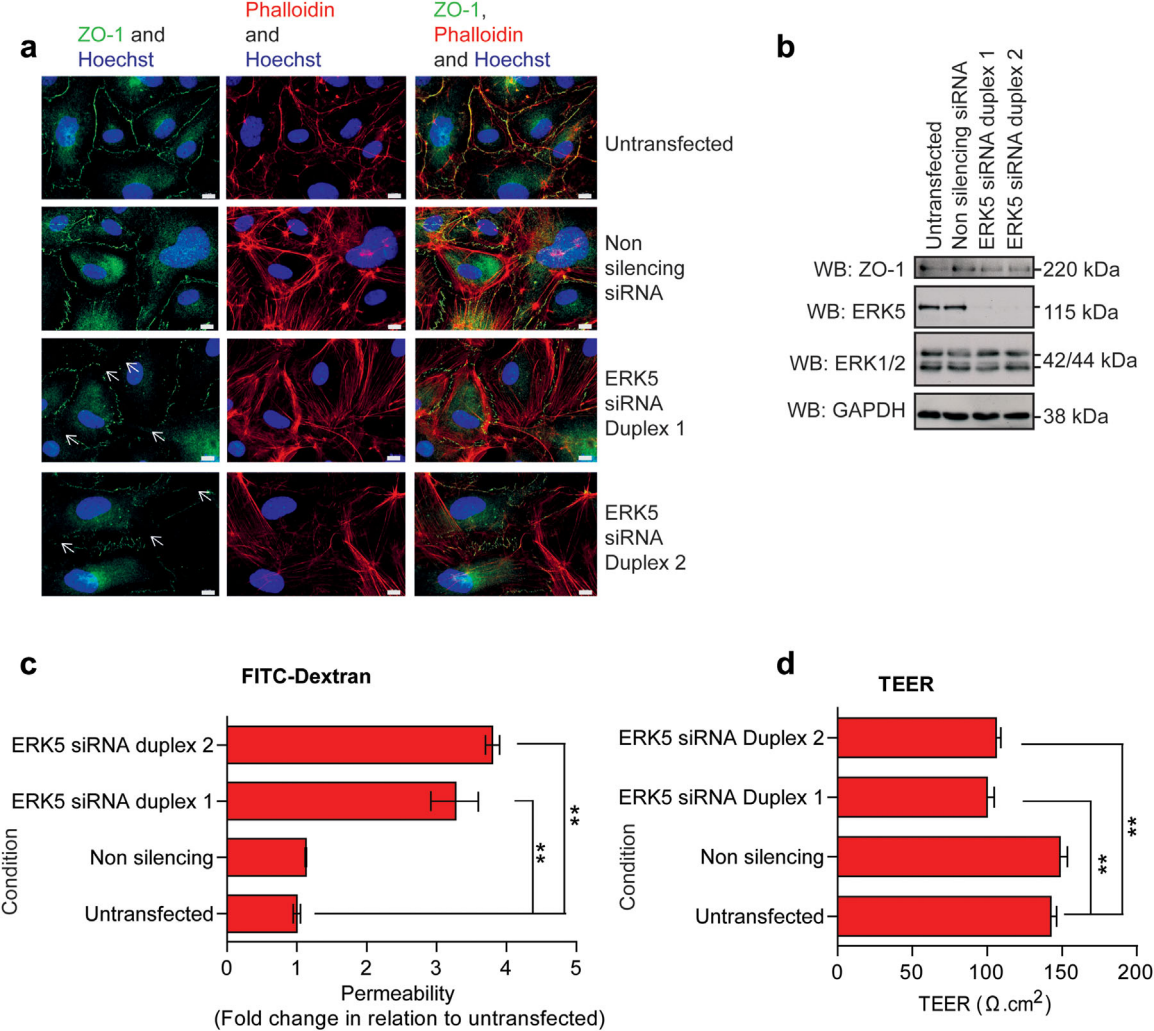
siRNA mediated gene silencing of ERK5 induced HCMEC barrier perturbment. HCMEC were transfected with non‐silencing or ERK5 siRNA for 6 hr and allowed to reach confluence over 5 subsequent days. (a) Immunofluorescence imaging of tight junctions (ZO‐1, green), actin stress fibers (phalloidin, red), and nuclei (Hoechst, blue). Arrows indicate barrier perturbment. Scale bars: 10 μm. Results are from one experiment representative of three. (b) Western blot of ZO‐1, ERK5, ERK1/2, and GAPDH levels in HCMECs following siRNA induced ERK5 gene silencing. (c) HCMECs were plated on Thincerts™ containing 0.4 μm pores and permeability of 4 kDa FITC‐dextran across the HCMEC monolayer was assessed and compared to untransfected cells (*n* = 4), mean ± s.d. **p ≤ 0.01 compared to untransfected. (d) Assessment of TEER was conducted on HCMECs plated on Thincerts™ containing 0.4 μm pores (*n* = 4), mean ± s.d. **p ≤ 0.01 compared to untransfected

Mouse *ERK5* knockout studies have suggested a role for ERK5 in preserving vascular integrity (Hayashi et al., 2004). Intercellular tight junctions are critical for endothelial cell barrier formation and regulation of paracellular diffusion (Tornavaca et al., 2015). We utilized siRNA‐mediated silencing of ERK5 to analyze the localization of the tight junction protein zonula occludens‐1 (ZO‐1) in human cardiac microvascular endothelial cells (HCMECs). ERK5 silencing resulted in reduced localization of ZO‐1 in tight junctions (Figure 1a) with no apparent effect on actin stress fiber formation analyzed by phalloidin staining (Figure 1a). The level of ZO‐1 protein did not appear to be affected by ERK5 silencing (Figure 1b). Loss of endothelial tight junction formation has been linked with an increase in paracellular permeability (Bazzoni & Dejana, 2004). Analysis of permeability using FITC‐labeled dextran (Figure 1c) revealed that ERK5 silencing resulted in an increase in permeability in HCMECs (Figure 1c). We also analyzed permeability by measuring transendothelial electrical resistance (TEER) (Wegener & Seebach, 2014). Treatment with ERK5 siRNA resulted in a significant decrease in TEER indicating increased permeability (Figure 1d). Taken together, these data suggest that ERK5 regulates endothelial tight junction formation and paracellular permeability.

**Figure 2:**
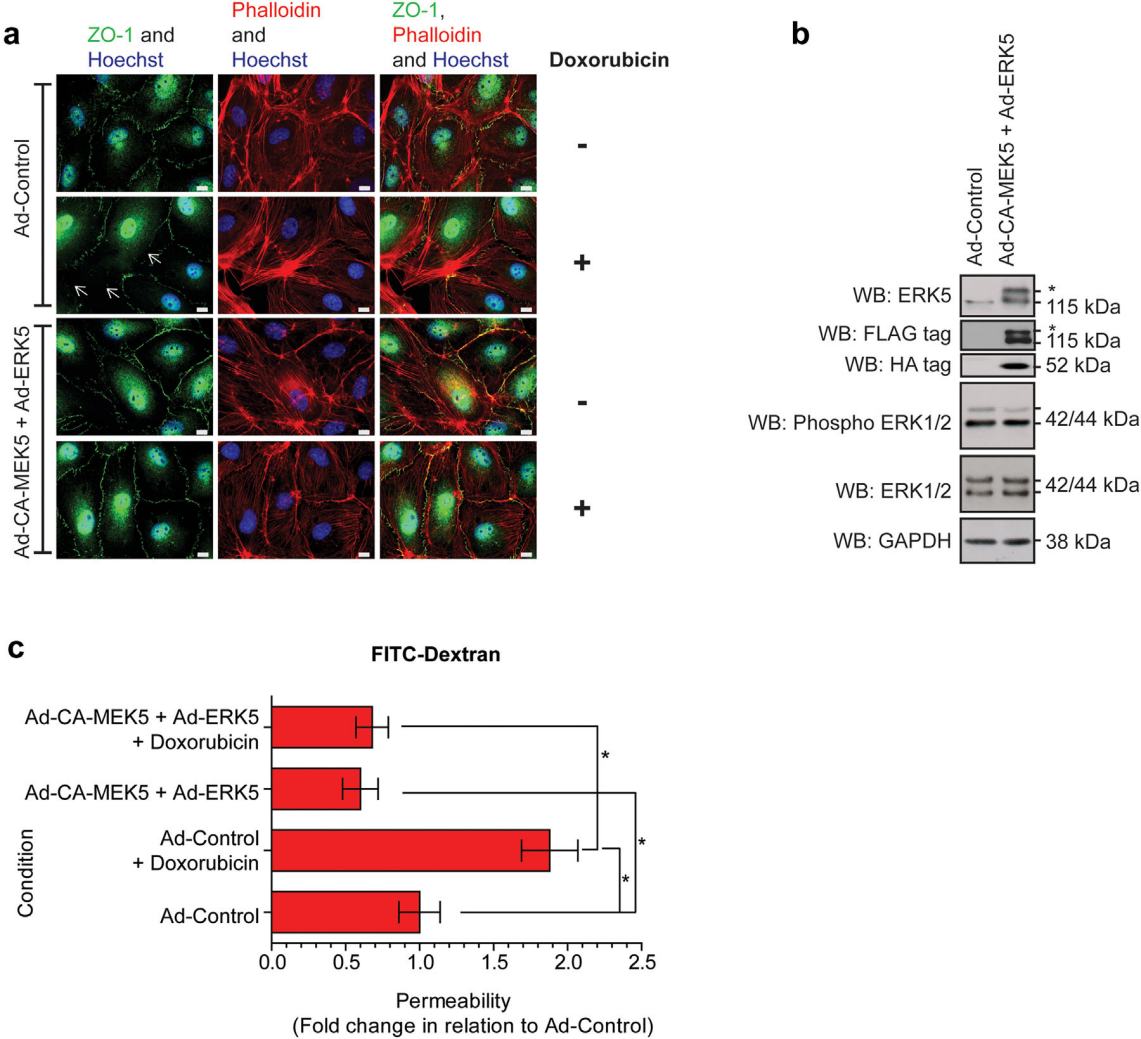
Adenoviral mediated ERK5 expression stimulates HCMEC tight junction formation. HCMEC were transfected with Ad‐Control or Ad‐CA‐MEK5 and Ad‐ERK5 for 24 hr before treatment with doxorubicin 0.1 μM for 6 hr. (a) Immunofluorescence imaging of HCMEC tight junctions (ZO‐1, green), actin stress fibers (phalloidin, red), and nuclei (Hoechst, blue). Arrows indicate barrier perturbment. Scale bars: 10 μm. Results are from one experiment representative of three. (b) Western blot of ERK5, ERK1/2, phospho ERK1/2, and GAPDH levels in HCMECs. FLAG‐Tag was used to confirm transfection of Ad‐ERK5 and HA‐Tag was used to confirm transfection of Ad‐CA‐MEK5. (c) HCMECs were plated on Thincerts™ with 0.4 μm pores and permeability of 4 kDa FITC‐dextran across the HCMEC monolayer assessed and compared to Ad‐Control (*n* = 4), mean ± s.d. *p ≤ 0.05 compared to Ad‐Control

We have previously shown that the cardiotoxic drug doxorubicin is able to disrupt tight junction formation in HCMECs resulting in increased permeability (Wilkinson et al., 2016). We used adenoviral mediated gene transduction to express a constitutively active MEK5 (CA‐MEK5) and ERK5 in HCMECs and analyze effects on tight junction formation and permeability. Expression of CA‐MEK5 and ERK5 resulted in increased tight junction formation and cortical actin staining, which was able to overcome the effects of doxorubicin (Figure 2a). Increased activation of ERK5 was confirmed by mobility shift on Western blot analysis (Figure 2b). Concomitant with the effects on tight junction formation, expression of CA‐MEK5 and ERK5 also resulted in decreased permeability in HCMECs (Figure 2c); this effect was able to prevent the increased permeability seen with doxorubicin treatment. These data show that increased ERK5 activity stimulates tight junction formation and decreases permeability in endothelial cells.

### 3.2 Statins induce ERK5 activation in HCMECs

**Figure 3:**
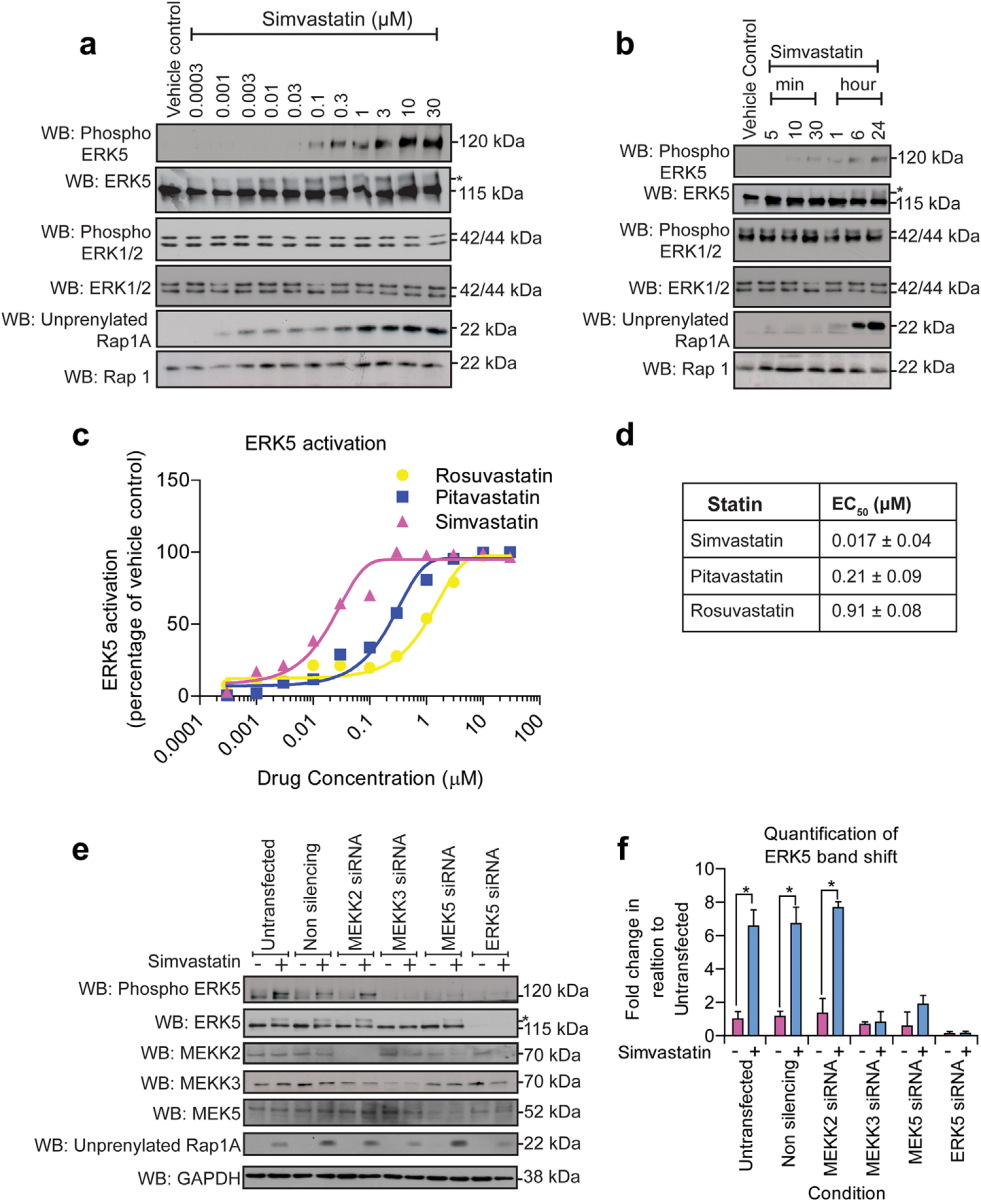
Simvastatin stimulates ERK5 phosphorylation in HCMECs. (a) HCMECs were incubated with simvastatin at a range of concentrations for 6 hr. Intracellular signaling responses were assessed by Western blotting for phosphorylation of ERK5 and ERK1/2 using phospho‐specific antibodies. Unprenylation of Rap1A was measured as a control for simvastatin activity. Total protein was measured for ERK5, ERK1/2, and Rap1. (b) HCMECs were incubated with 0.3 μM simvastatin at a range of time points. Intracellular signaling responses were assessed by Western blotting for phosphorylation of ERK5 and ERK1/2 using phospho‐specific antibodies. Unprenylation of Rap1 was measured as a control for simvastatin activity. Total protein was measured for ERK5, ERK1/2, and Rap1. (c) The level of ERK5 band shift following treatment with simvastatin, rosuvastatin, and pitavastatin was quantified relative to vehicle control. (d) Activation of ERK5 by band shift was determined (EC_50_ concentration), mean ± s.d. (*n* = 3). (e) HCMEC were transfected with siRNA to MEKK2, MEKK3, MEK5, and ERK5 for 6 hr and allowed to reach confluence over 5 subsequent days before treatment with 0.1% DMSO or 0.3 μM simvastatin for 6 hr. Western blot of ERK5, phosphorylated‐ERK5, MEKK2, MEKK3, MEK5, and GAPDH levels in HCMEC. (f) Quantification of ERK5 band shift (*n* = 3) mean ± s.d. *p ≤ 0.05 compared to untransfected basal

We have previously reported that VEGF is able to activate ERK5 in endothelial cells and regulate endothelial cell survival (Roberts et al., 2010). It has recently been reported that statin treatment of cells results in ERK5 activation (Chu, Duellman, Weaver, Tao, & Yang, 2015; Le et al., 2014). We were interested in determining the ability of statins to activate ERK5 in HCMECs. We initially utilized simvastatin (Zocor) to analyze effects on ERK5 and ERK1/2 in HCMECs. Treatment of cells with simvastatin for 6 hr resulted in a dose‐dependent activation of ERK5 in the absence of ERK1/2 activation (Figure 3a). To confirm the biochemical effect of statins in inhibiting HMG Co‐A reductase with a concomitant reduction in cholesterol and isoprenoid synthesis, we analyzed the prenylation status of the small MW GTP‐binding protein Rap1A by Western blotting (Antoine, Srivastava, Pirmohamed, & Park, 2010; Sidaway et al., 2004). Activation of ERK5 was only observed at time points where Rap1A was unprenylated (Figure 3a). A number of different statins with different pharmacokinetic properties have been generated over the last 15 years. Simvastatin is a lipophilic type I statin derived from a natural product (Gazzerro et al., 2012). We also utilized another type 1 statin pitavastatin (Livalo) as well as rosuvastatin (Crestor) a more recently developed synthetic type II hydrophilic statin (Schachter, 2005). All statins showed dose‐dependent activation of ERK5, with simvastatin the most potent with an EC_50_ of 17 nM (Figure 3c,d; Supplementary Figure S1). ERK5 is activated by phosphorylation of the T‐E‐Y motif by the upstream kinase MEK5 (MAPKK). MEK5 is activated by phosphorylation on Ser^311^/Thr^315^ by MEKK3 or MEKK2 (MAPKKK) (Chao, Hayashi, Tapping, Kato, & Lee, 1999; Sun et al., 2001). In order to determine if ERK5 was activated by simvastatin via the canonical pathway, we utilized siRNA duplexes to silence expression of MEKK2, MEKK3, MEK5, and ERK5. Silencing of MEKK3 and MEK5 reduced ERK5 activation as shown by a bandshift and phosphorylation of Thr^218^/Tyr^220^ (Figure 3e). Silencing of MEKK2 did not affect simvastatin‐mediated activation of ERK5. These data suggest that simvastatin treatment activates ERK5 in HCMECs via a pathway requiring MEKK3 and MEK5.

### 3.3 Statin induced ERK5 phosphorylation occurs via inhibition of protein geranylgeranylation

**Figure 4:**
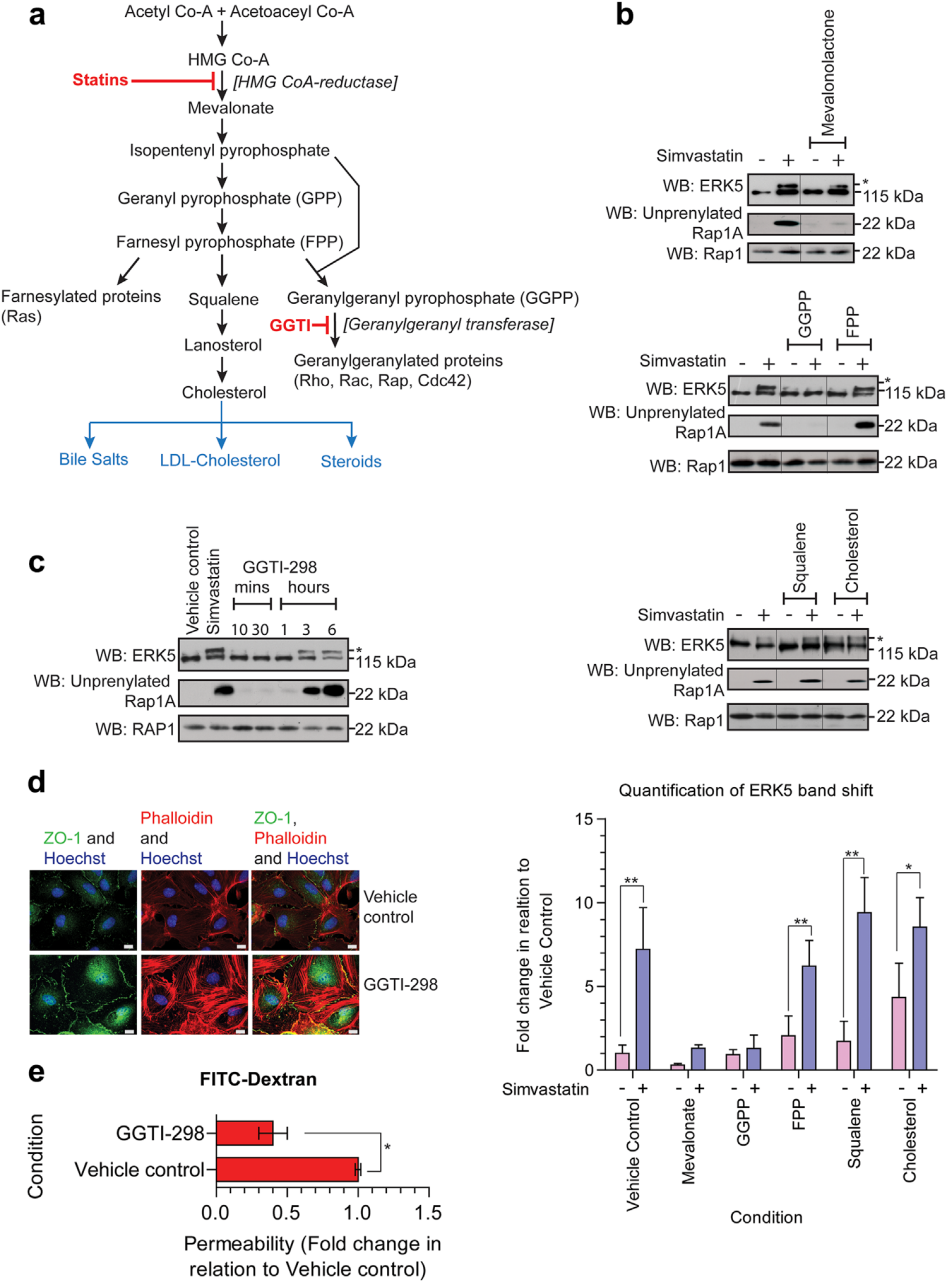
Statin induced ERK5 phosphorylation occurs via inhibition of protein geranylgeranylation. (a) Summary of the key substrates in the cholesterol biosynthesis pathway. HCMECs were treated with (b) mevalonolactone 50 μM, GGPP 10 μM, FPP 10 μM, squalene 10 μM, or cholesterol 10 μM for 24 hr in the presence and absence of simvastatin (0.3 μM for 6 hr). ERK5 expression was assessed by Western blotting. Unprenylation of Rap1A was measured as a control for simvastatin activity. The level of ERK5 activation assessed by mobility band shift was quantified and expressed as fold change relative to vehicle control (*n* = 4) mean ± s.d. *p ≤ 0.05, **p ≤ 0.01 compared to vehicle control basal. (c) HCMECs were treated with GGTI‐298 (10 μM) for a range of time points. Intracellular signaling responses were assessed by Western blotting for ERK5, unprenylated Rap1A and Rap1. (d) Immunofluorescence imaging of HCMEC tight junctions (ZO‐1, green), actin stress fibers (phalloidin, red), and nuclei (Hoechst, Blue) following treatment with GGTI‐298 (10 μM for 6 hr). Scale bars: 10 μm. (e) HCMEC were plated on Thincerts™ with 0.4 μm pores and permeability of 4 kDa FITC‐dextran across the HCMEC monolayer was assessed (*n* = 4), mean ± s.d. *p ≤ 0.05 compared to vehicle control

The previous results showed that simvastatin activation of ERK5 requires at least 6 hr pre‐incubation and occurs simultaneously with the unprenylation of Rap1A. Inhibition of HMG Co‐A reductase by statins leads to a reduction in mevalonate and the synthesis of isoprenoid intermediates and ultimately reduced cholesterol synthesis (Surani, Kimber, & Osborn, 1983) (Figure 4). In order to determine the biochemical pathway downstream of mevalonate reduction which leads to activation of ERK5, we used a series of biochemical add backs to determine at which point the effect of simvastatin on ERK5 activation could be prevented. Treatment with mevalonolactone, a membrane permeable ester of mevalonate (Vamvakopoulos & Green, 2003), prevented simvastatin‐stimulated ERK5 activation (Figure 4b). Treatment with cholesterol and the biochemical precursor squalene did not prevent simvastatin mediated activation of ERK5 (Figure 4b). Geranylgeranyl‐pyrophosphate (GGPP) and farnesyl‐pyrophosphate (FPP) are two isoprenoids generated from mevalonate. Treatment with cell‐permeable GGPP prevented simvastatin mediated ERK5 activation whereas treatment with cell permeable FPP had no effect (Figure 4b). Taken together, these data suggest that simvastatin activates ERK5 by suppressing the isoprenylation of proteins via GGPP. To further confirm the role of geranylgeranylation we utilized the geranylgeranyltransferase‐I inhibitor GGTI‐298 (Vogt, Qian, McGuire, Hamilton, & Sebti, 1996). This inhibitor activated ERK5 in HCMECs after 3 hr preincubation (Figure 4c). Furthermore, this inhibitor also stimulated tight junction formation (Figure 4d) and inhibited permeability in the HCMECs (Figure 4e).

### 3.4 Statins stimulate endothelial tight junction formation and decreased permeability via ERK5

**Figure 5:**
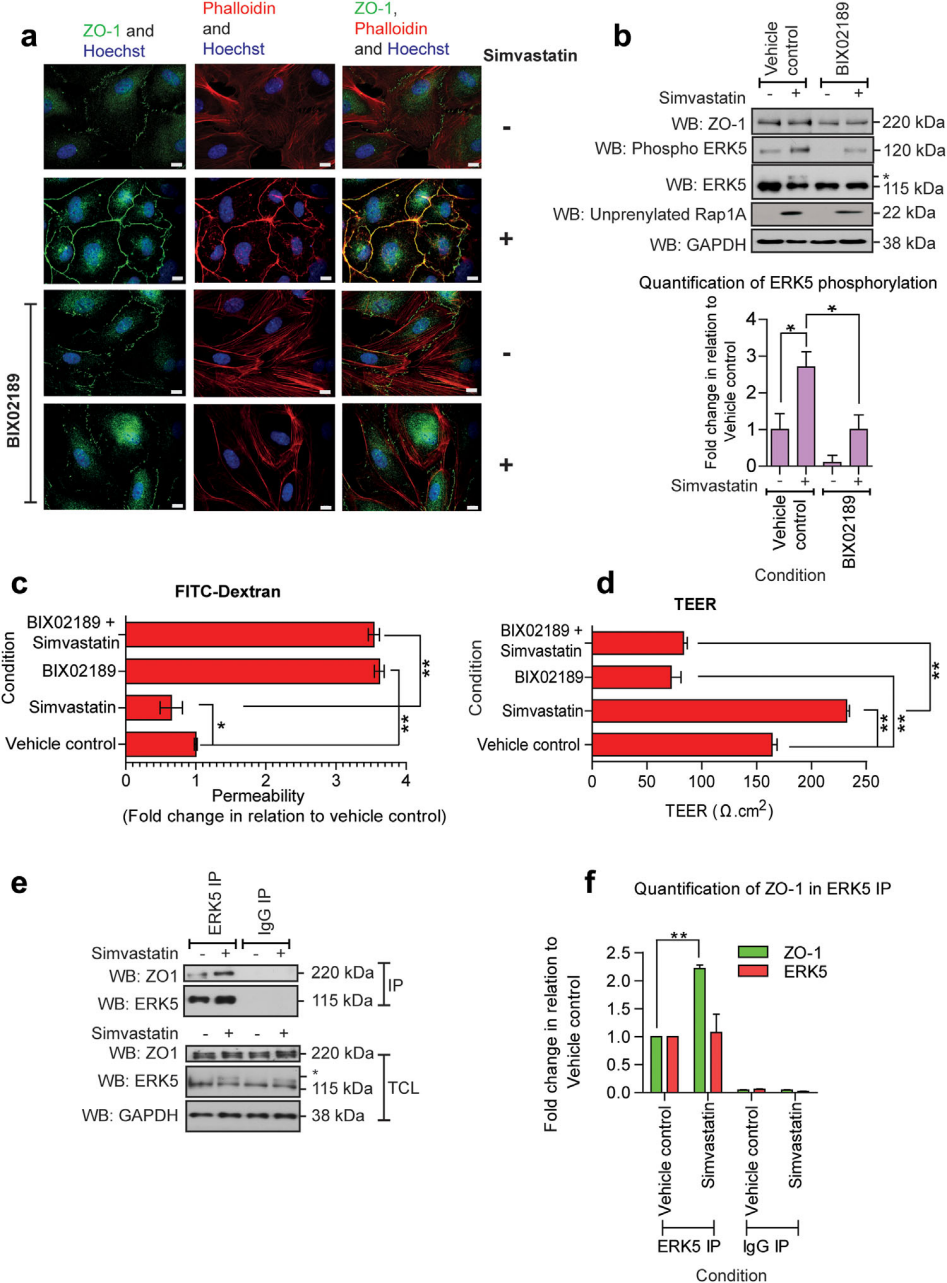
MEK5 inhibition prevents simvastatin induced tight junction formation and ERK5 phosphorylation. HCMECs were pre‐incubated with BIX02189 1 μM for 30 min priopr to addition of simvastatin 0.3 μM for 6 hr. (a) Immunofluorescence imaging of HCMEC tight junctions (ZO‐1, green), actin stress fibers (phalloidin, red), and nuclei (Hoechst, blue). Scale bars: 10 μm. Results are from one experiment representative of three. (b) Western blot of ZO‐1, ERK5, phospho ERK5, unprenylated Rap1A, and GAPDH levels in HCMECs. Level of ERK5 phosphorylation is quantified relative to vehicle control. Mean ± s.d. (*n* = 3) *p ≤ 0.05 compared to vehicle control basal. (c) Permeability of 4 kDa FITC‐dextran through an endothelial monolayer on ThinCerts™, (*n* = 4), mean ± s.d. *p ≤ 0.05, **p ≤ 0.01 compared to vehicle control. (d) Assessment of TEER was conducted on HCMECs plated on Thincerts™ containing 0.4 μm pores (*n* = 4), mean ± s.d. **p ≤ 0.01 compared to vehicle control. (e) HCMECs were treated with simvastatin 0.3 μM for 6 hr before immunoprecipitation with IgG or ERK5 antibodies. Western blot of ZO‐1 following immunoprecipitation with ERK5 in HCMECs. (f) Level of ZO‐1 and ERK5 is quantified relative to vehicle control in IP lysates. Mean ± s.d. (*n* = 3). **p ≤ 0.01, compared to vehicle control

Our initial data showed that constitutive activation of ERK5 can lead to increased endothelial tight junction formation and decreased permeability (Figure 2a–c). Statins have been shown to stimulate tight junction formation in human pulmonary artery endothelial cells (Chen et al., 2014) and rat brain endothelial cells (Morofuji et al., 2010). We were interested in determining if statins were also able to stimulate tight junction formation in an ERK5 dependent manner in HCMECs. Immunofluorescence analysis of ZO‐1 localization and actin stress fibers revealed that treatment of HCMECs with simvastatin for 6 hr resulted in increased ZO‐1 staining at the cell membrane and redistribution of actin stress fibers to form a cortical ring of F‐actin (Figure 5a). Preincubation with the MEK5 inhibitor BIX02189 (Tatake et al., 2008) prevented simvastatin‐mediated tight junction formation and actin reorganization (Figure 5a). The biochemical effect of BIX02189 in preventing ERK5 activation by simvastatin was confirmed by Western blotting (Figure 5b). Furthermore, the apparent increase in ZO‐1 staining at the plasma membrane following simvastatin treatment was not due to an increase in ZO‐1 protein levels (Figure 5b).

We have demonstrated that simvastatin significantly decreased endothelial permeability. This decrease in permeability was prevented by pretreatment with BIX02189 (Figure 5c,d). These data suggest that simvastatin regulated ERK5 activity leads to increased tight junction formation and reduced permeability in HCMECs. We next investigated the potential interaction between ZO‐1 and ERK5 following simvastatin treatment. Immunoprecipitation of ERK5 following simvastatin treatment for 6 hr resulted in co‐precipitation of ZO‐1 (Figure 5e,f) indicating that ERK5 and ZO‐1 can interact following simvastatin treatment.

### 3.5 Statin treatment prevents doxorubicin effects on tight junctions and cell permeability in HCMECs

**Figure 6:**
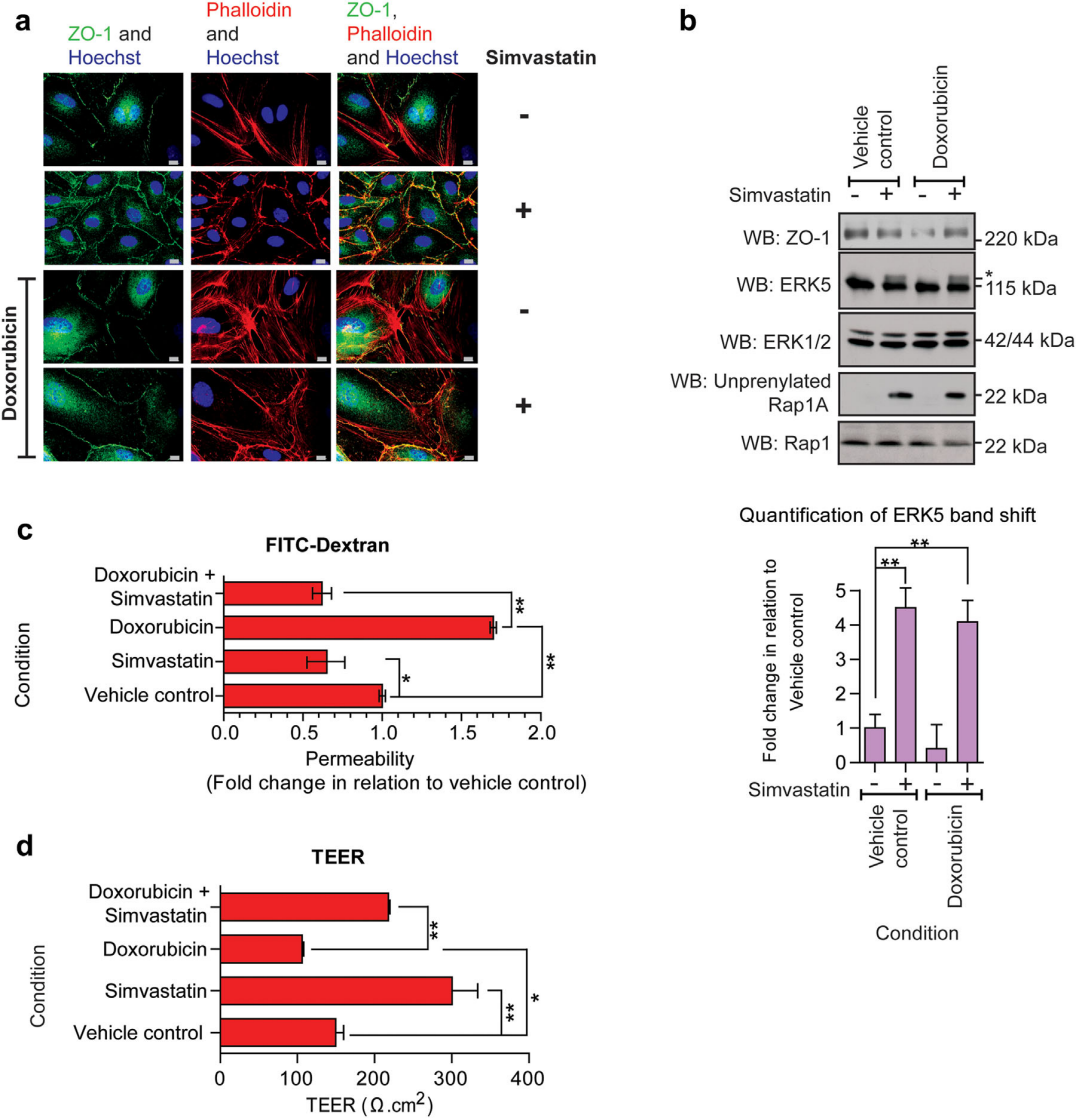
Simvastatin prevents doxorubicin induced barrier perturbment. HCMEC were pre‐incubated with simvastatin 0.3 μM for 6 hr before treatment with doxorubicin 0.1 μM for a further 6 hr. (a) Immunofluorescence imaging of HCMEC tight junctions (ZO‐1, green), actin stress fibers (phalloidin, red), and nuclei (Hoechst, blue). Scale bars: 10 μm. Results are from one experiment representative of three. (b) Western blot of ZO‐1, ERK5, ERK1/2, unprenylated Rap1A and Rap1 levels in HCMECs. Level of ERK5 activation is quantified relative to vehicle control. Mean ± s.d. (*n* = 3) **p ≤ 0.01 compared to vehicle control. (c) HCMEC were plated on Thincerts™ with 0.4 μm pores and permeability of 4 kDa FITC‐dextran across the HCMEC monolayer was assessed and compared to vehicle control (*n* = 4), mean ± s.d. *p ≤ 0.05, **p ≤ 0.01 compared to vehicle control. (d) Assessment of TEER was conducted on HCMECs plated on Thincerts™ with 0.4 μm pores (*n* = 4), mean ± s.d. *p ≤ 0.05, **p ≤ 0.01 compared to vehicle control

Statin treatment has been shown to reduce cardiotoxicity associated with doxorubicin treatment in vivo using rodent models (Henninger et al., 2015). We have already shown that adenoviral mediated overexpression of ERK5 in HCMECs can stimulate tight junction formation and overcome the effects of doxorubicin in HCMECs (Figure 2a–c). Considering that statins are able to activate ERK5 leading to increased tight junction formation, we determined the potential for statin pretreatment to overcome the adverse effect of doxorubicin on HCMECs. Pre‐treatment with simvastatin prevented doxorubicin‐induced disruption in tight junction formation (Figure 6a) and the concomitant increase in HCMEC permeability (Figure 6c,d). Simvastatin mediated activation of ERK5 was not affected by doxorubicin treatment (Figure 6b).

### 3.6 Statin treatment stimulates ERK5 translocation to the plasma membrane in HCMECs

**Figure 7:**
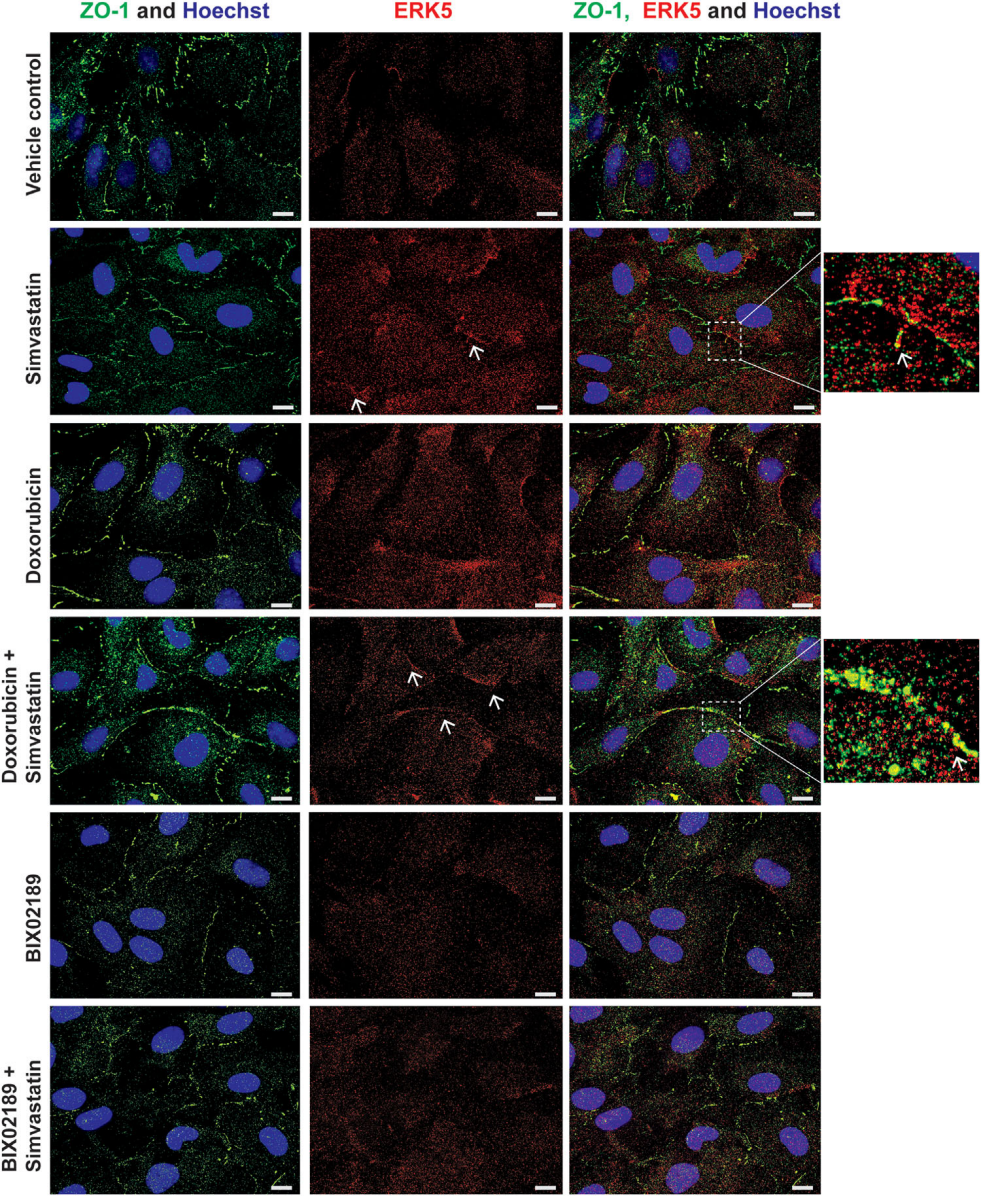
ERK5 and ZO‐1 colocalize following simvastatin treatment in HCMECs. Immunofluorescence imaging of HCMEC tight junctions (ZO‐1, green), ERK5 (red), and nuclei (Hoechst, blue) following treatment with simvastatin 0.3 μM and/or doxorubicin 0.1 μM or BIX02189 1 μM for 6 hr. Scale bars: 10 μm. Arrows indicate co‐localization. Results are from one experiment representative of three

**Figure 8:**
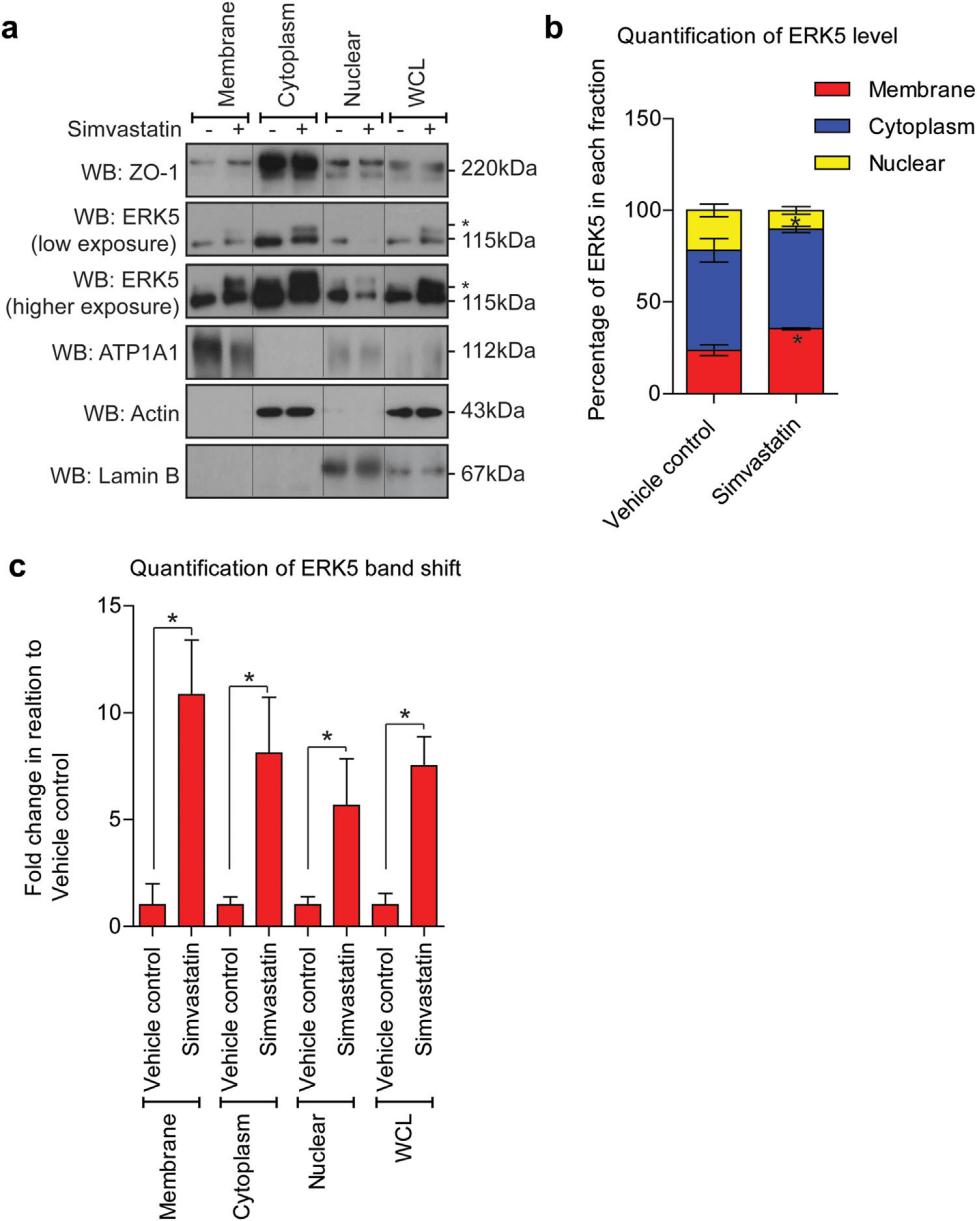
ERK5 cellular translocation following simvastatin treatment in HCMECs. HCMECs were treated with simvastatin 0.3 μM for 6 hr before subcellular fractionation performed. (a) Western blot of ZO‐1 and ERK5. ATP1A1, actin, and lamin B were used as positive controls to show cellular fraction specificity in membrane, cytoplasm, and nuclear fractions respectively. Whole cell lysate (WCL). (b) Quantification of the total ERK5 level in each compartment (*n* = 3), mean ± s.d. *p ≤ 0.05 compared to vehicle control for each fraction. (c) Quantification of ERK5 band shift relative to each fraction's vehicle control (*n* = 3), mean ± s.d. *p ≤ 0.05 compared to vehicle control for each fraction

Activation of ERK5 by phosphorylation on Thr^218^/Tyr^220^ residues in the kinase domain is thought to result in a conformational change that disrupts a nuclear export signal allowing translocation of ERK5 to the nucleus (Gomez, Erazo, & Lizcano, 2016; Kondoh, Terasawa, Morimoto, & Nishida, 2006) and regulation of MEF2 mediated gene expression (Kato et al., 1997). Analysis of ERK5 translocation by immunofluorescence revealed that in HCMECs, simvastatin stimulated significant ERK5 translocation to the plasma membrane, which appeared to show a degree of co‐localization with ZO‐1 (Figure 7). This co‐localization was maintained in the presence of doxorubicin, but was prevented by the MEK5 inhibitor BIX02189 (Figure 7). Analysis of simvastatin‐mediated ERK5 activation and translocation by subcellular fractionation revealed that increased ERK5 activation was evident in the membrane, cytoplasm, and nuclear fractions (Figure 8a,b) with ERK5 showing increased translocation to the membrane (Figure 8c).

## 4 DISCUSSION

Endothelial tight junctions maintain vascular integrity and regulate paracellular transport of molecules. In this study, we show that ERK5 regulates the tight junction adaptor protein ZO‐1 in endothelial cells. Pharmacological activation of ERK5 with statins results in increased association between ZO‐1 and ERK5 at the plasma membrane resulting in endothelial tight junction formation and decreased paracellular permeability. This effect can overcome both the perturbment of tight junction formation and increased permeability with doxorubicin.

The ERK5 signaling axis has been shown to play a critical role in maintaining vascular integrity in the cardiac circulation with *MEKK3*/*MEK5*/*ERK5* gene ablation in mice resulting in embryonic lethality at E9.5‐10.5 (Hayashi et al., 2004; Wang et al., 2005; Yang et al., 2000). The use of siRNA mediated gene silencing of ERK5 and overexpression of ERK5 in HCMECs revealed that ERK5 regulates the tight junction associated protein ZO‐1 and cell permeability (Figures 1 and 2). This finding was further supported by the increase in permeability observed with the MEK5 inhibitor BIX02189 (Figure 5c,d). Gene knockout of *ZO‐1* is embryonically lethal at E9.5‐10.5 with embryos displaying defective angiogenesis (Katsuno et al., 2008). Recent data analyzing ZO‐1 function in endothelial cells has shown that ZO‐1 is required for endothelial cell barrier formation and adherens junction formation (Tornavaca et al., 2015). Our data provide a novel link to explain why loss of *ERK5* results in disrupted vascular integrity and increased permeability in vivo.

Statins have been reported to stimulate a number of pleiotropic effects in endothelial cells independent of a reduction in serum cholesterol levels (Liao, 2005; Wolfrum, Jensen, & Liao, 2003). This vascular effect has been attributed in part to stimulation of endothelial nitric oxide synthase (eNOS) expression leading to vasodilation (Laufs, La Fata, Plutzky, & Liao, 1998). More recent data have shown that statin mediated activation of ERK5 in human umbilical vein endothelial cells (HUVECs) leads to induction of Kruppel‐like factor 4 (KLF4) and upregulation of eNOS expression (Ohnesorge et al., 2010).

In HCMECs, analysis of the kinetics of simvastatin mediated ERK5 activation revealed that activation of ERK5 occurred simultaneously with unprenylation of Rap1A (Figure 3). This suggests that activation occurs through inhibition of HMG Co‐A reductase, the pharmacological target of statins. The ability of cell permeable GGPP to prevent simvastatin activation of ERK5 (Figure 4b), suggested that loss of geranylgeranylation of specific GTP binding proteins, rather than loss of cholesterol synthesis, was responsible for activation of ERK5 in HCMECs. This was further confirmed by the use of the geranyltransferase I inhibitor GGTI‐298 (Figure 4c). A number of small GTP‐binding proteins require prenylation with geranylgeranyl groups to localise to biological membranes and allow interaction with downstream signaling molecules. These GTP‐binding proteins include Rho, Rac, Cdc42, and Rap (Takai, Sasaki, & Matozaki, 2001). Recent data have shown that atorvastatin treatment of HUVECs can stimulate endothelial barrier formation and also lead to increased cytosolic localization and activation of Rho, Rac, and Cdc42 (Xiao, Qin, Ping, & Zuo, 2013). Another report has shown that simvastatin treatment of bovine aortic endothelial cells (BAECs) can lead to increased activation of Rac (Kou, Sartoretto, & Michel, 2009). Our data show that statin activation of ERK5 in HCMECs requires MEKK3 (Figure 3e). It is plausible that inhibition of membrane localization and activation of small GTP‐binding proteins leads to activation of MEKK3, MEK5, and ultimately activation and translocation of ERK5 to the plasma membrane and interaction with ZO‐1 by an as yet undefined mechanism. Both simvastatin treatment of HCMECs and overexpression of constitutively active MEK5 resulted in ERK5 activation in the absence of ERK1/2 activation. Activation of ERK5 with agonists such as EGF in HeLa cells has been reported to result in a nuclear localization of ERK5 and transcriptional regulation (Kondoh et al., 2006). In contrast, in HCMECs, simvastatin treatment led to decreased nuclear translocation of ERK5 and increased membrane translocation (Figures 7 and 8). Statin mediated activation of ERK5, which occurs in the absence of other intracellular signaling pathways, such as ERK1/2, may allow translocation of ERK5 to the plasma membrane and regulation of tight junction formation and endothelial cell permeability.

The plasma *C*_max_ for simvastatin is approximately 19–30 nM (Bjorkhem‐Bergman, Lindh, & Bergman, 2011). Activation of ERK5 occurred at therapeutically relevant concentrations of simvastatin (Figure 3a–c). We found that within the statin class of drugs, the more lipophilic statins simvastatin and pitavastatin were more potent activators of ERK5 than the more hydrophilic rosuvastatin. This may reflect differential uptake of the drugs into HCMECs as lipophilic statins can be absorbed by passive diffusion through cell membranes (Schachter, 2005), whereas hydrophilic statins, such as rosuvastatin, have been shown to utilize the organic anion transporters (OAT) such as OATP1B1 encoded by the *SLCO1B1* gene, which is highly expressed in hepatocytes (Niemi, Pasanen, & Neuvonen, 2011). Analysis of *SLCO1B1* mRNA expression in HCMECs revealed very low expression of these transporters compared with liver tissue (Supplementary Figure S2). Analysis of statin uptake into different tissues in rodents has shown that the hydrophilic pravastatin inhibited sterol synthesis by 90% in the liver and ileum of rodents but less than 14% in kidney, spleen, adrenal, testis, prostate, and brain, whereas the lipophilic statins lovastatin and simvastatin inhibited this process in all tissues (Koga et al., 1990).

Recent studies in mice have shown that pretreatment with statins can protect against doxorubicin‐induced cardiotoxicity (Yoshida et al., 2009). This effect was attributed to direct effects on cardiomyocytes in a Rac‐dependent manner. Our data would suggest that the protective effect of statins can also occur at the level of the cardiac microvasculature via activation of ERK5 and increased tight junction formation with a concomitant decrease in cellular permeability of doxorubicin ultimately reducing exposure of cardiomyocytes to this drug. A clinical study in female breast cancer patients has shown that simultaneous use of statins and doxorubicin resulted in reduced heart failure compared with doxorubicin alone (Seicean, Seicean, Plana, Budd, & Marwick, 2012). Analysis of the effect of simvastatin on viability in HCMECs exposed to doxorubicin showed a potential protective effect in contrast to enhanced toxicity to doxorubicin in A2780 ovarian cancer cells and BT474 breast cancer cells (Supplementary Figure S3).

One potential drawback to the clinical use of statins in reducing cardiac vascular permeability and reducing cardiotoxicity is the potential reduced delivery of chemotherapeutic agent to the tumor, reducing clinical efficacy. However, an in vivo study in mice using three different human tumor xenografts has shown that lovastatin potentiates doxorubicin anti‐tumor activity while reducing doxorubicin‐associated cardiotoxicity (Feleszko et al., 2000). This would appear to back up our in vitro data. Furthermore, it is possible that statin mediated effects in potentially reducing tumor vascular permeability and delivery of drug to the tumor are ameliorated due to the inherent leakiness of tumor blood vessels compared with normal vasculature (Azzi, Hebda, & Gavard, 2013; De Bock et al., 2011).

In summary, our data show that ERK5 regulates tight junction formation and permeability in cardiac microvascular endothelial cells. Statin mediated ERK5 activation results in the translocation of ERK5 to the plasma membrane and regulation of tight junction formation and decreased endothelial cell permeability. Activation of ERK5 by drugs such as statins may have therapeutic potential in alleviating conditions where endothelial cell barrier formation is compromised such as drug‐induced cardiac injury (Wilkinson et al., 2016), arterial hypertension (Laine, 1988), sepsis (Rachoin, Cerceo, & Dellinger, 2013), and diabetic nephropathy (Peng et al., 2013).

## Supporting information

Additional Supporting Information may be found online in the supporting information tab for this article.

**Figure S1**. Statin induced ERK5 phosphorylation.

**Figure S2**. Transporter expression in endothelial cells in comparison to the liver.

**Figure S3**. Cell viability in response to doxorubicin in the presence and absence of simvastatin.
