# Supplementary figures and images for: Statin regulated ERK5 stimulates tight junction formation and reduces permeability in human cardiac endothelial cells
Source: J Cell Physiol. 2017 Aug 3;233(1):186–200. doi: 10.1002/jcp.26064 (PMC5655747; doi:10.1002/jcp.26064)

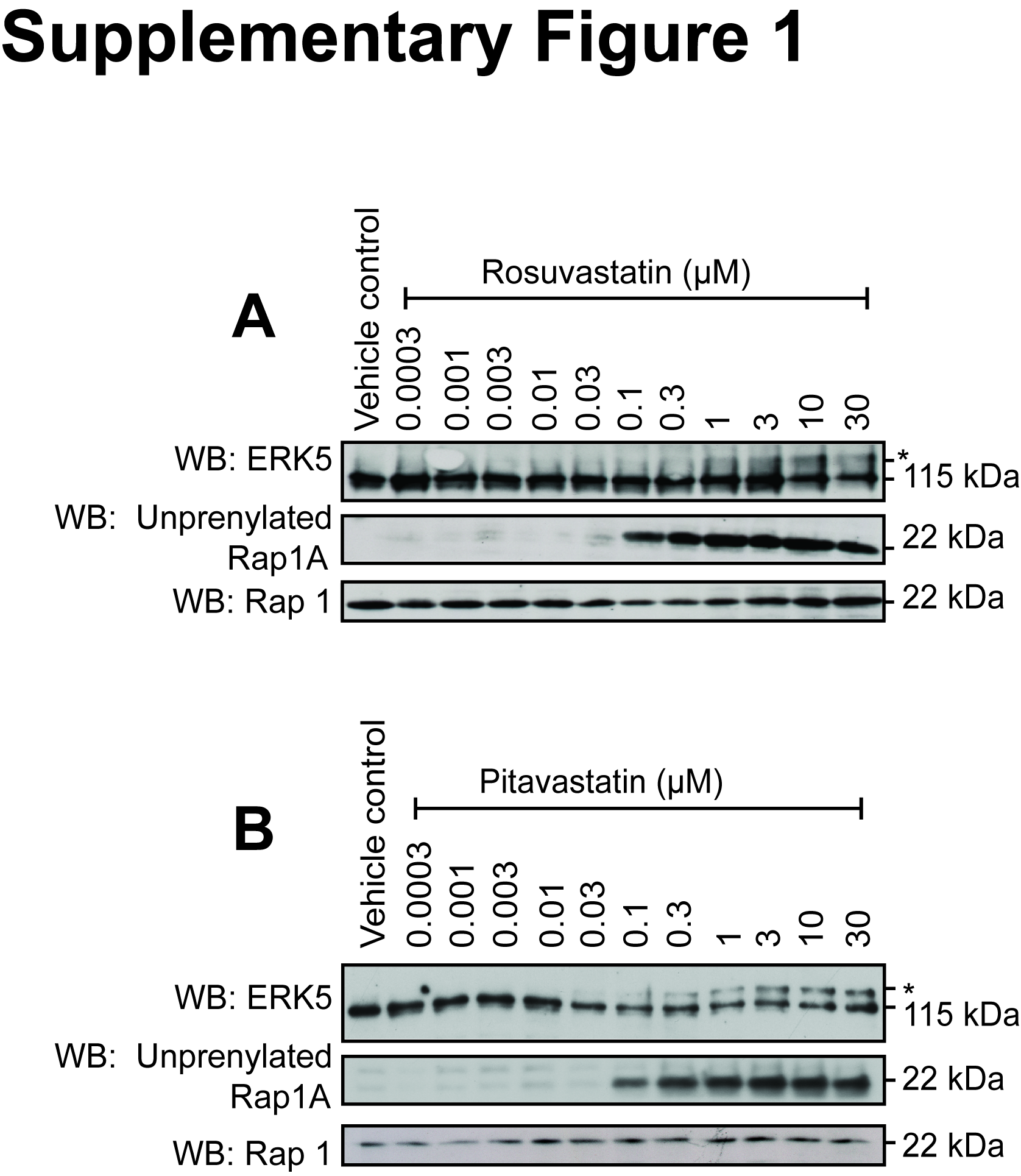

Supplement: Supplementary file 1 — Figure S1. Statin induced ERK5 phosphorylation. [file JCP-233-186-s001.tif]

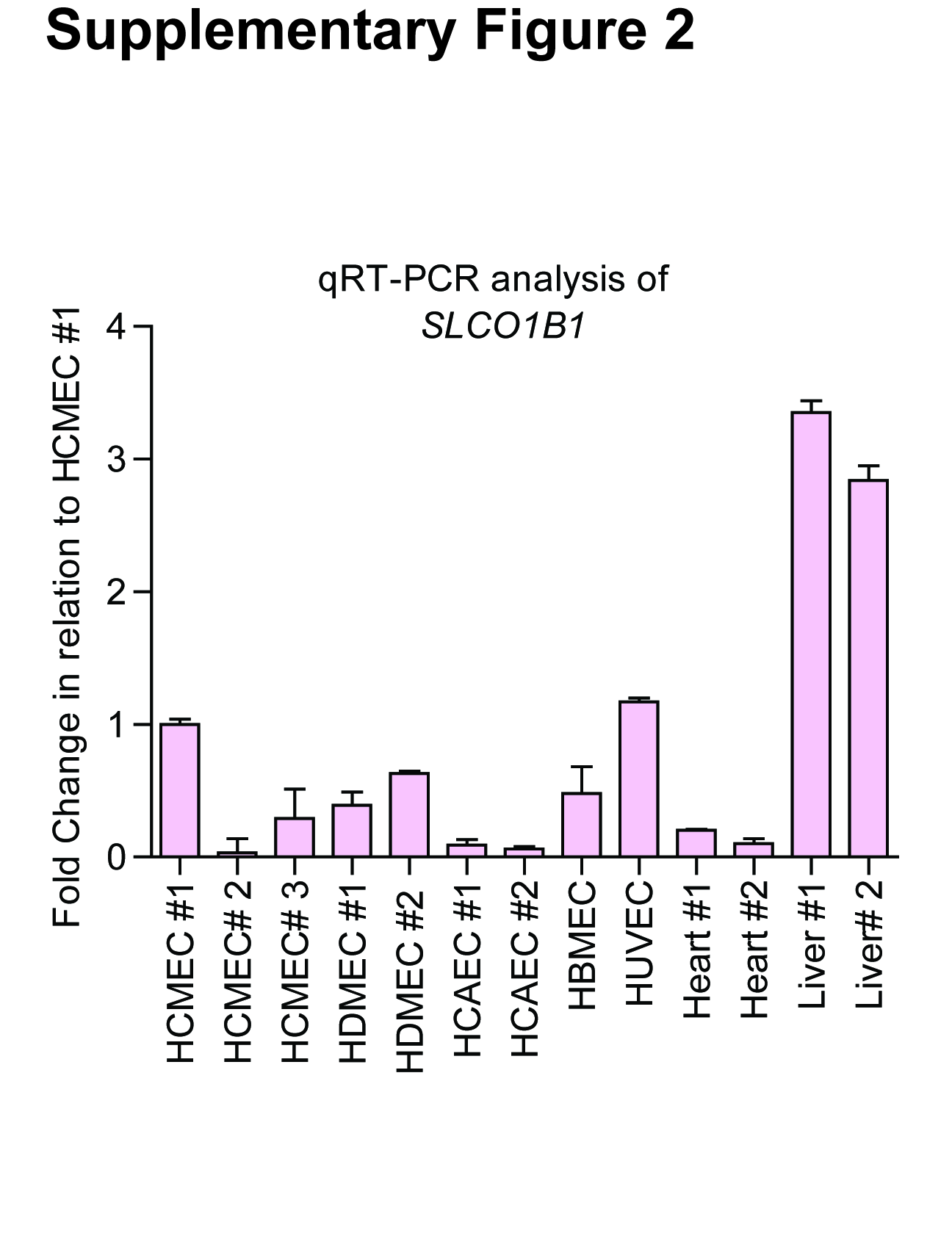

Supplement: Supplementary file 2 — Figure S2. Transporter expression in endothelial cells in comparison to the liver. [file JCP-233-186-s002.tif]

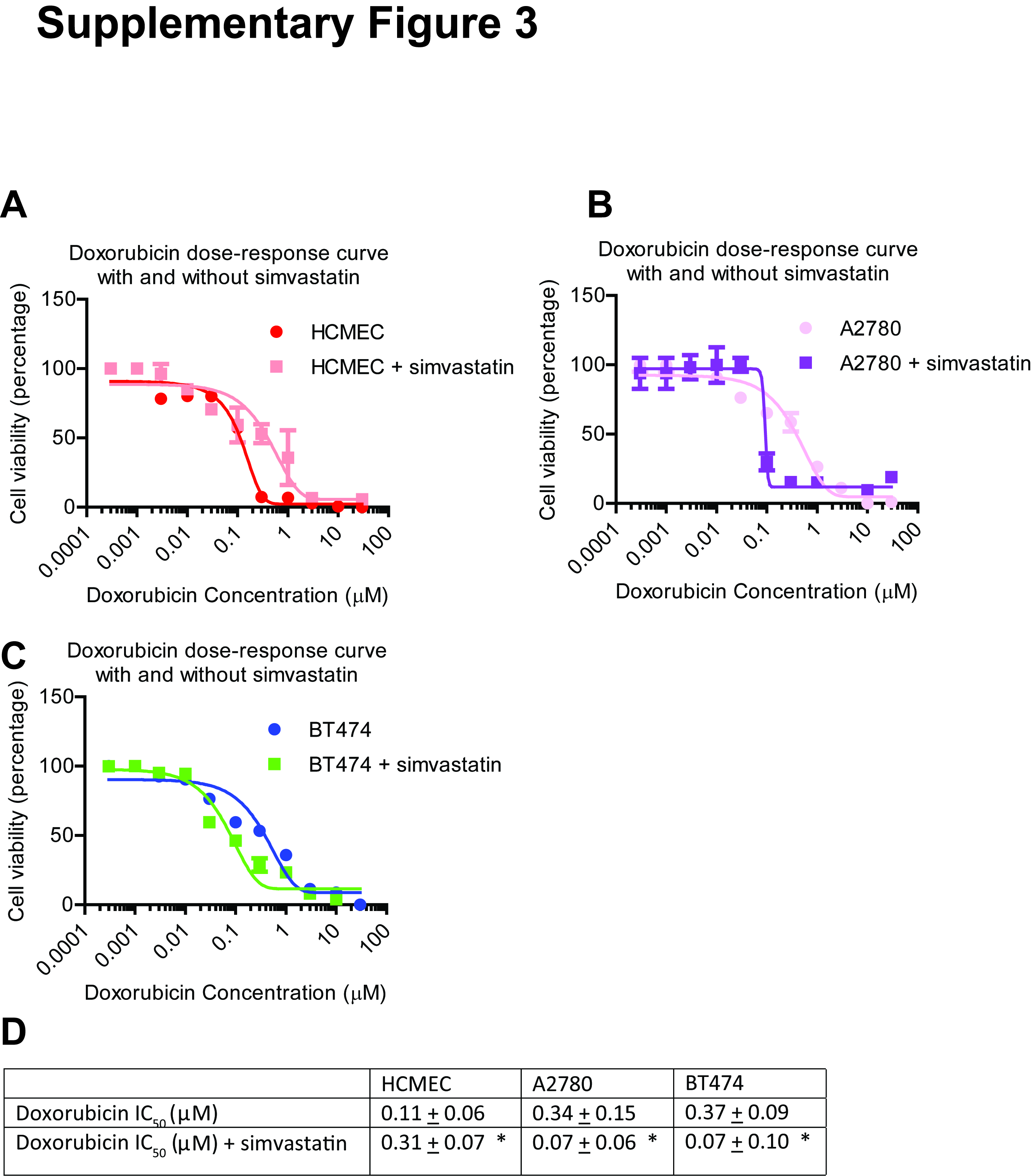

Supplement: Supplementary file 3 — Figure S3. Cell viability in response to doxorubicin in the presence and absence of simvastatin. [file JCP-233-186-s003.tif]
